# Dysfunction of basal ganglia functional connectivity associated with subjective and cognitive fatigue in multiple sclerosis

**DOI:** 10.3389/fnins.2023.1194859

**Published:** 2023-06-02

**Authors:** Christelle Langley, Naoki Masuda, Simon Godwin, Giovanni De Marco, Angela Davies Smith, Rosemary Jones, Jared Bruce, Ngoc Jade Thai

**Affiliations:** ^1^CRIC Bristol, Bristol Medical School, University of Bristol, Bristol, United Kingdom; ^2^Department of Psychiatry, University of Cambridge, Cambridge, United Kingdom; ^3^Department of Engineering Mathematics, University of Bristol, Bristol, United Kingdom; ^4^Department of Mathematics, State University of New York at Buffalo, Buffalo, NY, United States; ^5^Laboratoire CeRSM (EA-2931), UPL, Université Paris Nanterre, Nanterre, France; ^6^Bristol and Avon Multiple Sclerosis Centre, The Brain Centre, Southmead Hospital, Bristol, United Kingdom; ^7^Department of Biomedical and Health Informatics, University of Missouri – Kansas City School of Medicine, Kansas City, MO, United States; ^8^Mental Health Research for Innovation Centre, Mersey Care NHS Foundation Trust, Hollins Park House, Warrington, United Kingdom

**Keywords:** multiple sclerosis, fatigue, basal ganglia, neuroimaging, functional connectivity

## Abstract

**Objectives:**

Central fatigue is one of the most common symptoms in multiple sclerosis (MS). It has a profound impact on quality of life and a negative effect on cognition. Despite its widespread impact, fatigue is poorly understood and very difficult to measure. Whilst the basal ganglia has been implicated in fatigue the nature of its role and involvement with fatigue is still unclear. The aim of the present study was to establish the role of the basal ganglia in MS fatigue using functional connectivity measures.

**Methods:**

The present study examined the functional connectivity (FC) of the basal ganglia in a functional MRI study with 40 female participants with MS (mean age = 49.98 (SD = 9.65) years) and 40 female age-matched (mean age = 49.95 (SD = 9.59) years) healthy controls (HC). To measure fatigue the study employed the subjective self-report Fatigue Severity Scale and a performance measure of cognitive fatigue using an alertness-motor paradigm. To distinguish physical and central fatigue force measurements were also recorded.

**Results:**

The results suggest that decreased local FC within the basal ganglia plays a key role in cognitive fatigue in MS. Increased global FC between the basal ganglia and the cortex may sub serve a compensatory mechanism to reduce the impact of fatigue in MS.

**Conclusion:**

The current study is the first to show that basal ganglia functional connectivity is associated with both subjective and objective fatigue in MS. In addition, the local FC of the basal ganglia during fatigue inducing tasks could provide a neurophysiological biomarker of fatigue.

## 1. Introduction

Fatigue has a detrimental effect on everyday functioning (Chaudhuri and Behan, 2004; Boksem et al., 2005; Marcora et al., 2009; Faber et al., 2012). It is one of the most common symptoms in multiple sclerosis (MS), being reported in over 90% of patients, and is often reported as the most disabling symptom patient experience (Chaudhuri and Behan, 2000). It has a profound impact on quality of life, leads to loss of employment (Krupp et al., 1988; Janardhan and Bakshi, 2002) and has a negative effect on cognition (DeLuca, 2005; van der Hiele et al., 2015).

Despite the widespread impact of fatigue in MS, its underlying mechanisms remain unclear. This may be largely due to diagnostic ambiguity and lack of a clear definition. Some definitions include a sense of exhaustion, lack of energy, or tiredness (Krupp et al., 1988). Another definition states “subjective lack of physical and/or mental energy that is perceived by the individual or caregiver to interfere with usual or desired activity” (Multiple Sclerosis Council for Clinical Practice Guidelines, 1998). This definition highlights an important distinction between physical fatigue and cognitive fatigue. Physical fatigue is mostly seen in neuromuscular disorders and is more easily defined and measured than cognitive fatigue. A common definition is the reduction of the ability of a muscle to generate force accompanied by an increase in the perceived effort by the individual (Bigland-Ritchie et al., 1978; Liu et al., 2005). Physical fatigue can result from increased exertion, or a reduction or complete failure in the neuromuscular system resulting in an inability to generate force (Enoka and Stuart, 1992). Physical fatigue can be measured indirectly through electromyography (EMG) and directly from force measurements. On the other hand, cognitive fatigue has been defined as the enhanced perception of mental effort and limited endurance for sustained physical and mental activities (Chaudhuri and Behan, 2004). There is no standardized method for measuring cognitive fatigue. Previous studies have measured cognitive fatigue as a decrease in performance (increased errors and increased reaction time) during sustained mental effort (Boksem et al., 2005, 2006; Moeller et al., 2012). One study operationalized cognitive fatigue as increased cerebral activation (DeLuca et al., 2008). Moreover, there has often been a lack of correlation between subjective self-report measures and performance measures of cognitive fatigue (Bailey et al., 2007), demonstrating the difficulty in measuring cognitive fatigue.

Neuroimaging techniques have been employed in an attempt to elucidate the neural substrates of fatigue. Research has suggested (Chaudhuri and Behan, 2000, 2004) that the non-motor functions of the basal ganglia, through the loss of motivational influence from the striato-thalamic system to the frontal lobes, may be involved in cognitive fatigue. Two studies that have experimentally examined cognitive fatigue in MS both show support for this model and implicate the basal ganglia in MS fatigue (DeLuca et al., 2008; Finke et al., 2015). However, the studies demonstrated opposing effects of the basal ganglia. DeLuca et al. (2008) used a modified version of the Symbol Digit Modalities Test (SDMT) during functional magnetic resonance imaging (fMRI). The authors suggested that in a healthy population brain activity should decrease over sustained task performance. This may be due to a variety of factors such as practice effects (Raichle et al., 1994), a switch from controlled to automatic processing (Koch et al., 2006), priming (Vuilleumier et al., 2002) or habituation (Bandettini et al., 1997). Whereas, patients with MS show widespread increased activation in the brain, which may be due to increased effort (Chiaravalloti et al., 2005). Therefore, DeLuca et al. (2008) operationalized cognitive fatigue as increased cerebral activation in the MS group compared to controls. Results showed hyperactivity of the basal ganglia, supporting the basal ganglia’s involvement in cognitive fatigue. The authors posited that increased cerebral activation may be due to a compensatory neural mechanism rather than cognitive fatigue *per se*. The authors were unable to distinguish between the two explanations because they did not have a measure of subjective fatigue.

In contrast to DeLuca et al. (2008) task-based fMRI, Finke et al. (2015) performed resting-state fMRI in 44 MS patients. They reported that fatigue severity, as measured with the self- report Fatigue Severity Scale (FSS), where increased fatigue was associated with lower functional connectivity (FC) of basal ganglia with medial prefrontal cortex, precuneus and posterior cingulate cortex in MS patients. Therefore, decreased connectivity of BG and prefrontal cortex may contribute to fatigue pathophysiology in MS. This finding appears to contradict the DeLuca et al. (2008) finding that hyperactivity of basal ganglia is associated with fatigue. The differences between Finke et al. (2015) and DeLuca et al. (2008) could be due to the former being resting state fMRI with only a subjective measure of fatigue and latter a task-based fMRI study using a performance-based measure of cognitive fatigue.

In the present study we used both an objective and subjective measure of fatigue to better elucidate the involvement of the basal ganglia in MS fatigue. Our objective measure was an alertness-motor paradigm involving prolonged alertness, which has been demonstrated to induce fatigue (Boksem et al., 2005; Oken et al., 2006; Faber et al., 2012). In addition, this paradigm allowed for us to examine cognitive and physical fatigue separately. Our measure of subjective fatigue was the Fatigue Severity Scale (FSS) (Krupp et al., 1988) completed by the participants. We examined both the global FC between the basal ganglia and the cortex, and, for the first time, the local FC within the basal ganglia. If hyperactivity of the basal ganglia were associated with cognitive fatigue, we would expect to observe increased connectivity between the basal ganglia and the cortex in fatigued groups. Conversely if hypoactivity of basal ganglia were related to cognitive fatigue, we would expect to observe decreased connectivity between the basal ganglia and the cortex in fatigued groups.

## 2. Methods

### 2.1. Participants

40 females with a diagnosis of MS (mean age = 49.98 (SD = 9.65) years) were recruited from The Brain Centre at Southmead Hospital. Diagnoses were made by a consultant neurologist according to the McDonald Criteria (McDonald et al., 2001; Polman et al., 2005). 40 female age-matched HC (mean age = 49.95 (SD = 9.59) years) were recruited from the community. The present study used only females for two reasons, (1) there are a large amount of sex differences in the attention literature (Der and Deary, 2006; Jain et al., 2015; Riley et al., 2016) and (2) MS affects significantly more females then males, with an approximate ratio of 3:1 (Wallin et al., 2019). MS participants were included if they scored 36 and above on the FSS (Krupp et al., 1988), whereas HC were excluded if they scored 36 or above. Participants were excluded if they showed any contraindications for MRI, were left-handed (participants confirmed they were predominantly right-handed), or if the MS participants had excessive upper limb tremor. Moreover, due to the high concordance between fatigue and mental health participants with comorbidities were excluded. This was measured as a score above 12 for either subsection, on the Hospital Anxiety and Depression Scale (Zigmond and Snaith, 1983). Ethical approval was granted by the Frenchay Research Ethics Service (16/SW/0059). The data is not available for sharing as no ethical or participant consent was obtained to share data.

In the MS group disease duration ranged between 1 and 36 years with a mean of 12.6 years (9.33 SD). Physical functioning was measured by the RAND Short Form-36 Health Survey 10 item sub-scale on physical functioning (SF-36) was generally high (17.28 mean, 5.19 SD).

### 2.2. Alertness-motor paradigm

**Figure 1:**
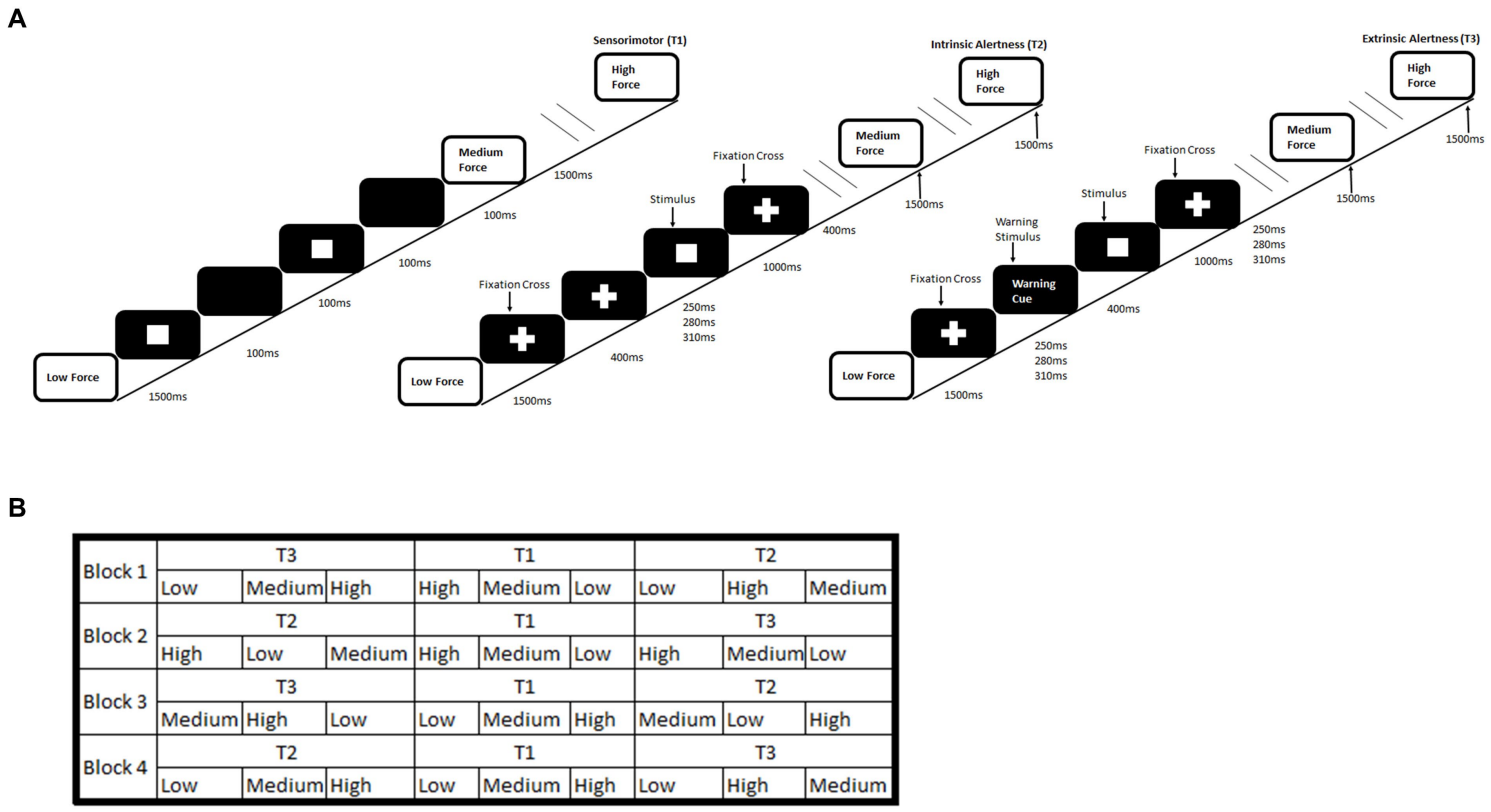
Schematic of an alertness-motor paradigm. **(A)** shows the task schematic where during the sensorimotor (T1) participants are required to respond to the flashing square and continuously squeeze the handgrip at the required force. For the intrinsic alertness task (T2) participants must respond at the required force to the white square and a central fixation cross was displayed permanently. For the extrinsic alertness task participants must respond at the required force to the white square, but a warning stimulus is presented for 400 ms prior to the stimulus appearing. **(B)** shows the interleaved task order.

The alertness-motor paradigm has been previously used and validated for fMRI used (Daly et al., 2020). The paradigm consisted of interleaved blocks of three different tasks, sensorimotor (T1), intrinsic alertness (T2) and extrinsic alertness (T3). Each task was repeated four times. The order of tasks was pseudorandomised. In the sensorimotor task (T1) participants were asked to squeeze and release the handgrip at their own pace. This was used as a control task. During the intrinsic alertness task (T2) the participants were asked to respond as soon as they saw the white square appear. They were told that there would be no warning cue and they were required to constantly maintain alertness. For the extrinsic alertness task (T3) participants were again instructed to squeeze as soon as they saw the white square appear but during this task, they were told that a warning cue would appear prior to the square. Therefore, they would know when the white square was going to appear. A schematic of the complete paradigm is shown in Figure 1.

Intrinsic alertness (T2) is defined as the internal control of attention and occurs without a warning signal. It represents a self-motivated state of awareness (Plohmann et al., 1998; Sturm et al., 1999). In contrast, extrinsic alertness represents the ability to increase the response readiness following the presence of a warning stimulus (Posner, 1975; Plohmann et al., 1998) where the presence of the warning signal (T3) produces a decrease in reaction time (Perin et al., 2010; Petersen and Posner, 2012). Our study required participants to perform both alertness tasks and to make their responses using a hand dynamometer at different levels of force. This unique paradigm allowed us to measure both physical fatigue and cognitive fatigue. Physical fatigue was operationally measured as decreased force over the duration of the paradigm and cognitive fatigue was operationally measured as reduction in performance through increased reaction time over the duration of the paradigm, similar to previous studies (Boksem et al., 2005, 2006; Moeller et al., 2012).

Furthermore, we split the participants into two groups; handgrip only group and mental imagery first group. In the handgrip condition, participants performed the full paradigm whilst squeezing a hand dynamometer quickly, for approximately 1 s, at either a low, medium or high force. In the mental imagery condition, participants performed the full paradigm but were asked to only imagine squeezing the hand dynamometer at the required force. The mental imagery first group completed a mental imagery condition of the task, prior to the full handgrip condition. The addition of the mental imagery task prior to the handgrip task would create a group who had undergone a longer period of sustained mental effort. This could produce two possible effects, a practice effect, similar to DeLuca et al. (2008), where we would expect to see a reduced reaction time in the mental imagery first group. Alternatively, a fatiguing effect, where we would expect to see an increased reaction time in the mental imagery first group, due to the longer period of sustained mental effort. Nineteen HC and 19 MS participants were in the handgrip only group. Seventeen HC and 18 MS participants comprised the mental imagery first group.

### 2.3. Behavioural analysis

The reaction time of each participant was calculated as the difference between the onset of the force grip and the onset of the white square. Cognitive fatigue was operationally defined as an increased reaction time over the duration of the task, specifically between block 1 and block 4 of the task (Boksem et al., 2005, 2006; Moeller et al., 2012). A 2×2 ANOVA was conducted to compare between HC and MS groups (between group), for each of the two tasks (within group). Participants were split into two groups depending on whether they completed the imagery or handgrip only. Paired *t*-tests for the HC and MS groups, were used to determine differences within group performance across the task.

Physical fatigue was operationally defined as a decreased force over the duration of the task. Independent sample t-tests were conducted to compare between the HC and MS groups. Further paired t-tests were used to determine differences within group performance (handgrip only or mental imagery first group) across the task (block1 vs. block 4), for HC and MS groups separately.

We conducted Pearson’s correlation between subjective FSS scores, cognitive fatigue (reaction time) and physical fatigue (high force). Pearson’s correlation were also used to examine whether basal ganglia FC was associated with measures of subjective fatigue (FSS), cognitive fatigue (reaction time), and physical fatigue (high force). Analyses were conducted in SPSS 24 and the threshold of *p* < 0.05 was used.

### 2.4. Force measurements

The force exerted was detected and transduced by the hand dynamometer, this apparatus was attached to a radiofrequency filter which enabled the data to be transferred from the scan room to the DA100C transducer amplifier. The transducer amplifier was connected to a MP150 device which allowed the force exerted by the participant to be transformed into an optical signal and visualised on the software acknowledge on a PC in the MRI control room. The force measurement was calculated as the average peak force for each participant during each block.

### 2.5. Image acquisition

MRI scans were performed in a 3 Tesla Siemens Skyra Magnetom scanner using 32 channel radiofrequency head coil. Two fMRI scans, for each condition, were acquired using T2*-weighted multiband gradient echo planar sequence (Moeller et al., 2010). Each scan lasted approximately 15 min. Thirty-nine slices that were orientated parallel to the anterior–posterior commissure plane and covered the whole brain were acquired. The parameters for the functional scans were: time to repetition (TR): 906 ms; time to echo (TE): 30 ms; field of view (FoV): 192 mm; voxel size: 3 × 3 × 3 mm and a multiband acceleration factor of 3. T1-weighted inversion recovery magnetisation prepared rapid acquisition gradient echo (MPRAGE) was acquired in the sagittal plane, comprising of 192 slices; TR: 1800 ms; TE: 2.25 ms; 9 mm isotropic voxel; and FoV of 240 mm for co-registration with functional scans. Furthermore, this MPRAGE image was used for the segmentation of grey matter, white matter and cerebral-spinal fluid for noise reduction in the connectivity analysis.

### 2.6. Pre-processing

All the functional images were pre-processed in SPM12.^1^ Images were realigned and resliced; coregistered and segmented to normalise images into standard space based on the MNI template, and Gaussian smoothing using 8 mm full-width half-maximum (FWHM) Gaussian kernel. The default SPM12 steps were used, except during the normalisation step, where the voxel size was set to 2 × 2 × 2 and the bounding box was changed. This was done to ensure that the data matched the automated anatomical labelling atlas (AAL) (Tzourio-Mazoyer et al., 2002) used for definition of ROIs and that all ROIs would be consistent across participants. The AAL is a brain parcellation atlas based on anatomical brain regions, it contains a total of 116 regions. Following the pre-processing steps, noise from white matter, cerebrospinal fluid and movement signals were regressed out using least squares multiple regression, from each voxel. A bandpass filter (0.01–0.08) was applied to remove low and high-frequency noise. A mean time series was then extracted from each of the 116 ROIs. The time series were split according to the timings of each task (T1,T2,T3) and then concatenated to represent each of the tasks in the paradigm. The FC between ROIs was measured using Pearson’s correlation, resulting in a 116×116 weighted connectivity matrix for each participant. To increase the normality and standardise the data for group comparison a Fisher z-transform was conducted. These standardised weighted connectivity matrices were used for the network analysis, performed in MATLAB (2015a) (Mathworks Inc., Natick, MA, United States).

### 2.7. Network analysis

The local FC of the basal ganglia was estimated by computing the average connectivity between the right and left caudate and the right and left putamen (Caudate-Putamen), the average connectivity between the right and left caudate and the right and left pallidum (Caudate-Pallidum) and finally the average connectivity of the right and left putamen and the right and left pallidum (Putamen-Pallidum). The global FC was computed as the average connectivity between all other 110 ROIs and the caudate (left and right combined), the putamen (left and right combined), the pallidum (left and right combined) and an average basal ganglia connectivity of all 6 basal ganglia regions. We applied the Benjamini and Hochberg (1995) procedure for the false discovery rate (FDR) to the FC of the basal ganglia, due to the number of comparisons. The threshold was set *a priori* at *q* < 0.10. To compare between MS and HC groups, independent sample t-tests were conducted for the local and global FC of the basal ganglia.

To examine the impact of fatigue, paired t-tests for basal ganglia global FC were conducted. Participants were split into two groups; handgrip only and mental imagery first group. This was to examine the differences between the first and last block of the alertness tasks in both HC and MS groups. The local basal ganglia FC was correlated, using Pearson’s correlation, with subjective fatigue (FSS), cognitive fatigue (reaction time) and physical fatigue (high force).

## 3. Results

### 3.1. Behavioural results

Reaction time outliers defined by 2 standard deviations above and below the mean were removed due to incorrect task performance. The final sample consisted of 37 MS participants (aged 35–67, mean 50.11, SD 9.40) and 36 HC (aged 31–68, mean 49.69, SD 9.95).

### 3.2. Cognitive fatigue

**Figure 2:**
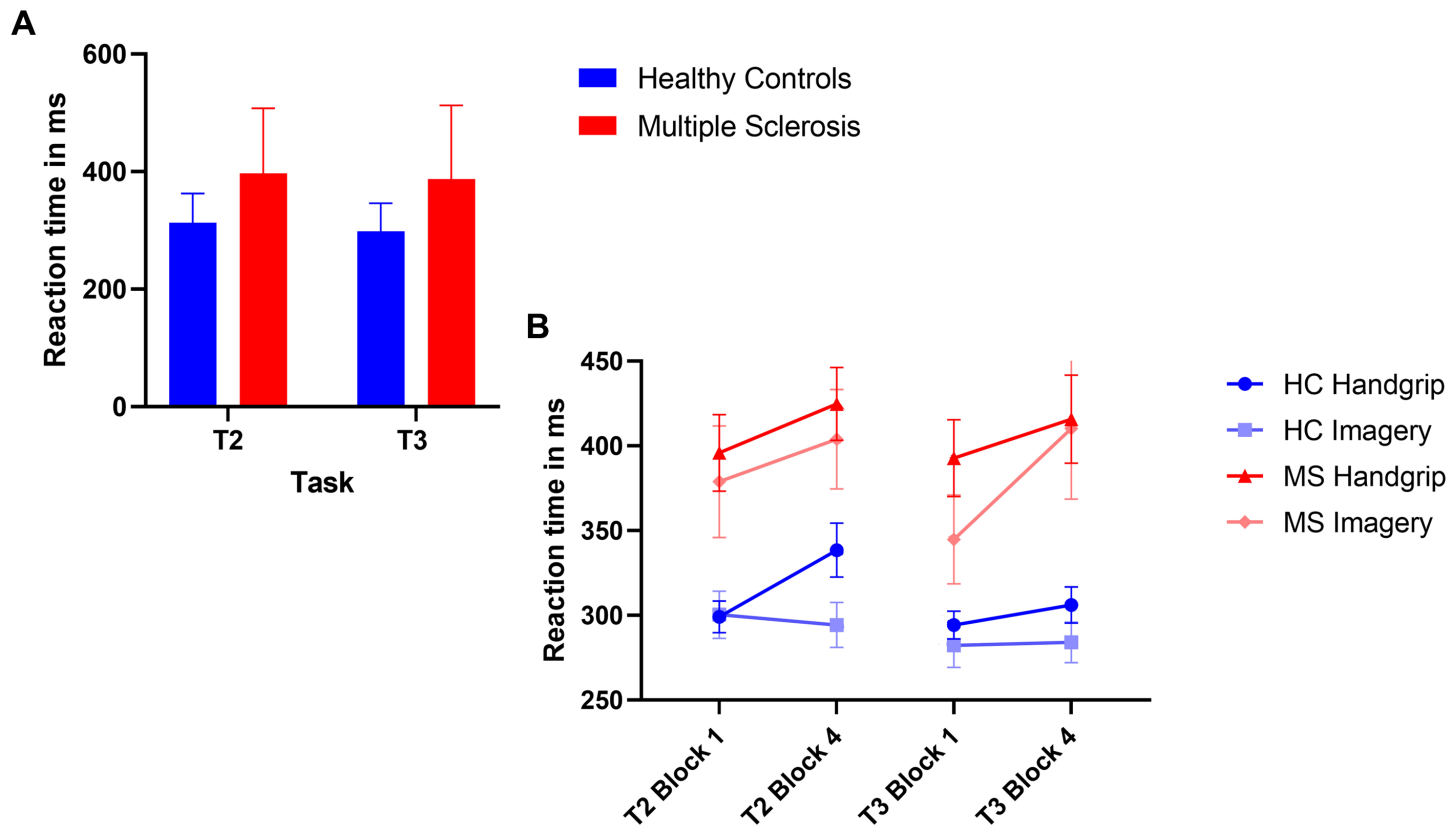
Composite image of reaction time performance. **(A)** Represents global task reaction time performance, where the HC group are displayed in blue and the MS are displayed in red. **(B)** Represents the reaction time performance at the beginning (block 1) and end (block 4) of the task as split by condition order. The HC groups are in shades of blue and the MS group are in shades of red. The error bars represent the standard error of the mean.

The 2×2 ANOVA revealed a significant main effect of task (*F*(1,71) = 5.45, *p* = 0.02, η_p_^2^ = 0.71), where there was reduced reaction time in the extrinsic alertness task, displayed in Figure 2A. There was a significant main effect of diagnosis (F(1,71) = 18.18, *p* < 0.01, η_p_^2^ = 0.20), where the patients with MS performed significantly slower than the HC group in both the intrinsic and extrinsic alertness tasks. There was no significant interaction effect between task and diagnosis (F(1,71) = 0.45, *p* = 0.51, η_p_^2^ = 0.01).

The analysis of reaction time performance in the HC group showed that the HC handgrip only group showed increased cognitive fatigue, an increased reaction time in block 4 of T2 compared to block 1 (*t*(18) = −3.69, *p* < 0.01). There were no reaction time differences for this group during T3 (*t*(18) = −1.07, *p* = 0.30). The HC mental imagery first group showed no evidence of cognitive fatigue and reaction times remained stable for the duration of T2 (*t*(16) = 0.39, *p* = 0.70) and T3 (*t*(16) = −0.18, *p* = 0.86).

The MS handgrip only group showed evidence of cognitive fatigue as the reaction times significantly increased in the final block of T2 compared to the first block (*t*(18) = −2.55, *p* = 0.02). There was no reaction time difference for T3 (*t*(18) = −1.80, *p* = 0.09). The MS mental imagery first group exhibited the opposite pattern of results, no evidence of cognitive fatigue in T2 (*t*(17) = −1.47, *p* = 0.16) but a significantly increased cognitive fatigue during T3 (*t*(17) = −2.41, *p* = 0.03) was observed. Reaction times are displayed in Figure 2B.

The Pearson’s correlations showed a strong positive correlation between subjective fatigue (FSS) and cognitive fatigue (reaction time) (T2 RT R = 0.476, *p* < 0.01; T3 RT R = 0.38, *p* = 0.02), see Table 1. In addition, there was no correlation between age and FSS scores in either the MS (*R* = −0.03, *p* = 0.86) or HC (*R* = 0.19, *p* = 0.25) groups. Significant results survived FDR at *q* < 0.10.

**Table 1:** Pearson’s correlations for local FC of the basal ganglia and measures of fatigue.

			FSS	T2 RT	T3 RT	T2 Force	T3 Force
		FSS		0.476**	0.38*	−0.20	−0.10
Intrinsic alertness	HC	Caudate-Putamen	−0.22	−0.19	−0.16	−0.08	−0.06
		Caudate-Pallidum	−0.24	−0.21	−0.18	−0.10	−0.07
		Putamen-Pallidum	−0.36*	−0.38*	−0.20	−0.12	−0.08
	MS	Caudate-Putamen	−0.23	−0.21	−0.18	−0.11	−0.08
		Caudate-Pallidum	−0.24	−0.22	−0.19	−0.13	−0.10
		Putamen-Pallidum	−0.38*	−0.42**	−0.30	−0.20	−0.16
Extrinsic alertness	HC	Caudate-Putamen	−0.19	−0.18	−0.13	−0.07	−0.02
		Caudate-Pallidum	−0.21	−0.20	−0.14	−0.09	−0.02
		Putamen-Pallidum	−0.28	−0.25	−0.18	−0.06	−0.05
	MS	Caudate-Putamen	−0.21	−0.18	−0.15	−0.09	−0.05
		Caudate-Pallidum	−0.22	−0.21	−0.17	−0.11	−0.06
		Putamen-Pallidum	−0.30	−0.29	−0.21	−0.13	−0.09

HC *n* = 36; MS *n* = 37, * *p* < 0.05, ** *p* < 0.01. HC = healthy control, MS = multiple sclerosis, FSS=Fatigue Severity Scale, T2 = intrinsic alertness, T3 = extrinsic alertness, RT = reaction time.

### 3.3. Physical fatigue

**Figure 3:**
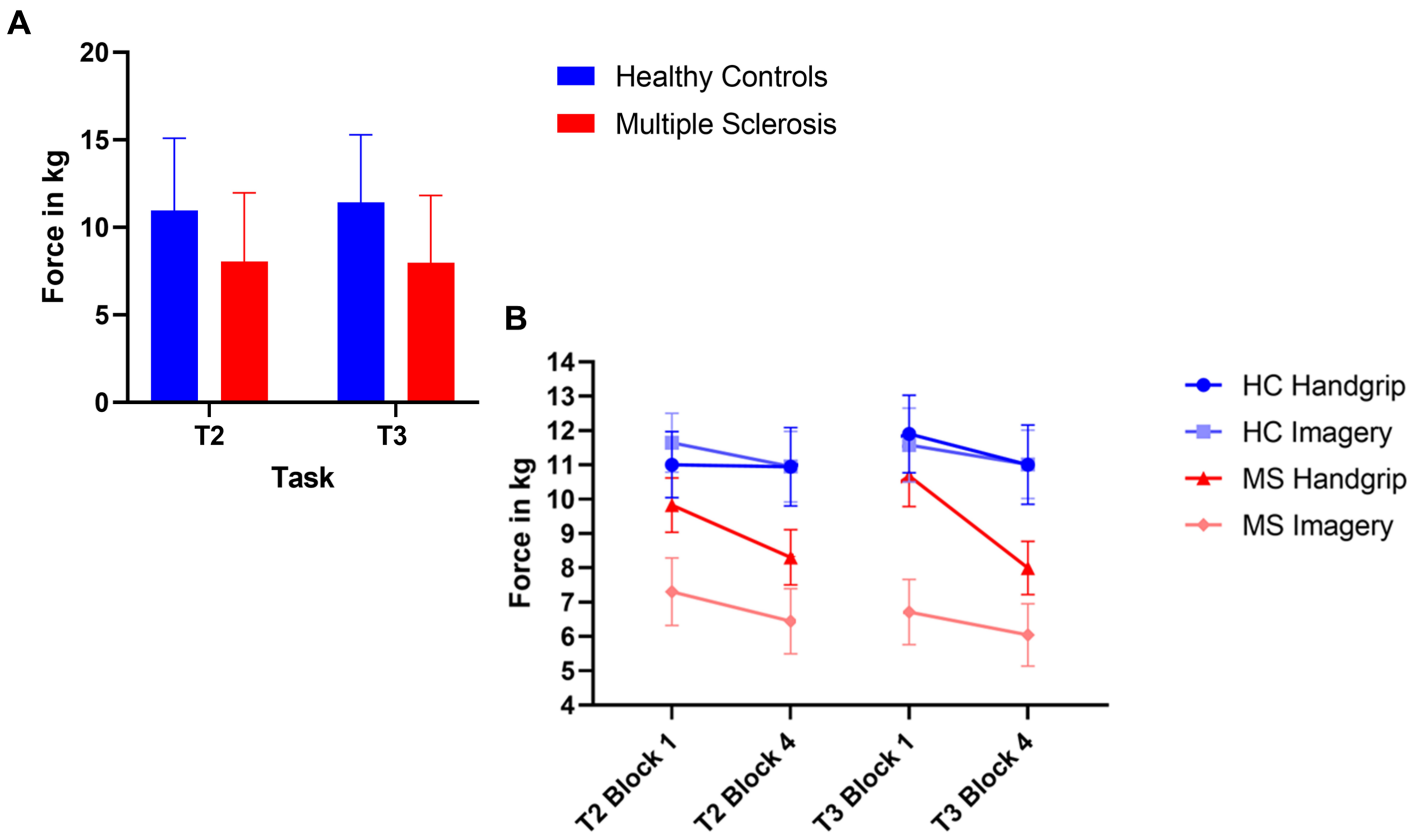
Composite image of force performance. **(A)** Represents global task performance in the high force condition, where the HC group are displayed in blue and the MS are displayed in red. **(B)** Represents the force performance in the high force condition at the beginning (block 1) and end (block 4) of the task as split by condition order. The HC group are in shades of blue and the MS group are in shades of red. The error bars represent the standard error of the mean.

The MS group had significantly decreased force grip compared to the HC group for high force during both intrinsic alertness task (*t*(71) = 3.09, *p* < 0.01, *d* = 0.72) and extrinsic alertness task (*t*(71) = 3.80, *p* < 0.01, *d* = 0.89). The force grip for each grip strength in both groups is displayed in Figure 3A.

The HC handgrip only group showed no significant differences for high force during the intrinsic alertness task (high M_1_ = 11.01, M_4_ = 10.95, *p* = 0.64), or the extrinsic alertness task (M_1_ = 11.90, M_4_ = 11.01, *p* = 0.25). Similarly, the HC group that completed the mental imagery first group showed no differences in force between block 1 and 4 of either the intrinsic alertness task (high M_1_ = 11.65, M_4_ = 10.95, *p* = 0.42) or extrinsic alertness task (high M_1_ = 11.58, M_4_ = 11.02, *p* = 0.38).

The MS handgrip only group showed no significant differences for high force during the intrinsic alertness task (high M_1_ = 9.83, M_4_ = 9.31, *p* = 0.42), or the extrinsic alertness task (M_1_ = 10.68, M_4_ = 9.76, *p* = 0.14). Similarly, the MS group that completed the mental imagery first showed no differences in force between block 1 and 4 of either the intrinsic alertness task (high M_1_ = 7.30, M_4_ = 6.85, *p* = 0.51) or extrinsic alertness task (high M_1_ = 6.71, M_4_ = 6.05 *p* = 0.47). Force performance is displayed in Figure 3B.

The Pearson’s correlations (Table 1) showed no association between subjective fatigue (FSS) and physical fatigue (force) during either the intrinsic alertness task or the extrinsic alertness task.

### 3.4. Network results

#### 3.4.1. Local basal ganglia functional connectivity

The comparison between HC and MS groups revealed a significant decreased local FC between the putamen and the pallidum in the MS group for both the intrinsic alertness (*t*(71) = 3.15, *p* < 0.01, d = 0.74) and the extrinsic alertness tasks (*t*(71) = 2.54, *p* < 0.01, *d* = 0.59). Full results are displayed in Table 2.

**Table 2:** Local basal ganglia functional connectivity for HC and MS groups during performance of an alertness-motor paradigm.

Task	Connectivity	HC		MS				
		Mean	SD	Mean	SD	t value	df	Cohens D
Intrinsic alertness	Caudate-Putamen	0.25	0.15	0.30	0.23	−0.86	71	0.20
	Caudate-Pallidum	0.28	0.14	0.30	0.21	−0.55	71	0.13
	Putamen-Pallidum	1.21	0.21	1.05	0.21	3.15**	71	0.74
Extrinsic alertness	Caudate-Putamen	0.29	0.19	0.29	0.17	−0.12	71	0.02
	Caudate-Pallidum	0.27	0.17	0.30	0.17	−0.68	71	0.16
	Putamen-Pallidum	1.18	0.21	1.05	0.23	2.54**	71	0.59

HC *n* = 36; MS *n* = 37, * *p* < 0.05, ** *p* < 0.01. HC = healthy control, MS = multiple sclerosis, SD = standard deviation, df = degrees of freedom.

**Figure 4:**
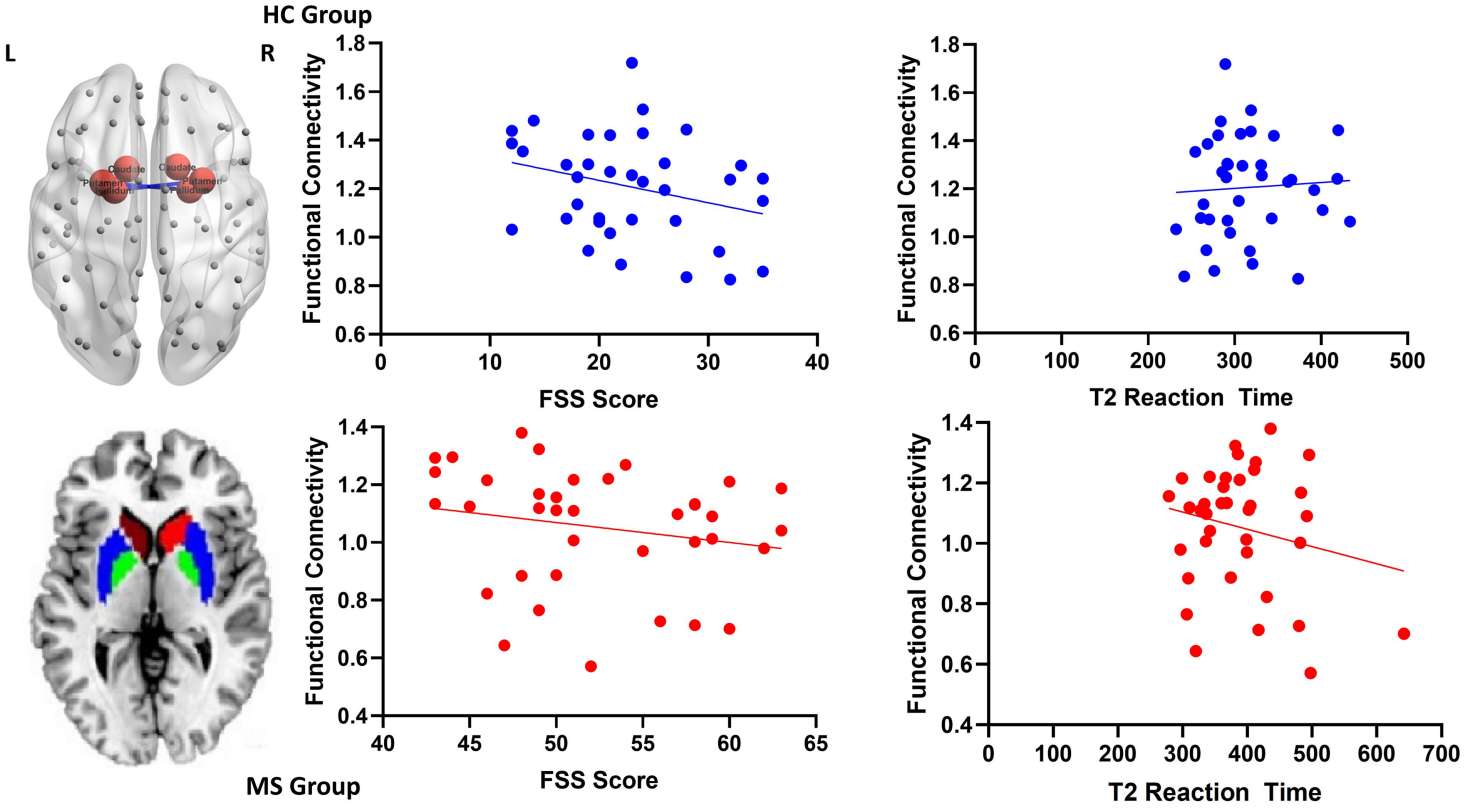
Scatterplots for local functional connectivity between the putamen and the pallidum. On the top left is an axial schematic of all the nodes in the AAL template, with the basal ganglia highlighted in red and the connection between the putamen and pallidum in blue. On the bottom left is an axial schematic of the basal ganglia regions where the caudate is red, the putamen is blue and the pallidum is green. On the right are scatterplots between putamen-pallidum FC and both FSS scores and intrinsic alertness reaction time for both the HC group, displayed in blue, and the MS group, displayed in red.

Increased local FC between the putamen and the pallidum during the intrinsic alertness task was associated with lower subjective fatigue (FSS) in both HC (*R* = −0.36, *p* = 0.03) and MS (*R* = −0.38, *p* = 0.02) groups. Similarly, increased local FC between the putamen and the pallidum during the intrinsic alertness task was associated with less cognitive fatigue during the intrinsic alertness task for both the MS (*R* = −0.42, *p* = 0.01) and HC groups (*R* = −0.38, *p* = 0.02) (Figure 4). All correlations are displayed in Table 1.

There were no significant correlations between subjective, cognitive or physical fatigue measures for either the HC or MS group with local FC between the putamen and the pallidum during the extrinsic alertness task.

#### 3.4.2. Global basal ganglia functional connectivity

The comparison between HC and MS groups revealed a significant increase in global FC of the putamen to the rest of the cortex in the MS group during the extrinsic alertness task (*t*(58.7) = −2.48, *p* = 0.01). There were no significant differences between the groups during the intrinsic alertness task (Table 3).

**Table 3:** Global basal ganglia functional connectivity for the split HC groups during the intrinsic alertness task.

Task	Connectivity	Block 1		Block 4			
		Mean	SD	Mean	SD	*t* value	df
Handgrip only	Caudate	0.38	0.15	0.39	0.18	−0.22	18
	Putamen	0.37	0.13	0.38	0.13	−0.04	18
	Pallidum	0.37	0.12	0.40	0.18	−0.68	18
	Average	0.37	0.09	0.39	0.14	−0.46	18
Mental imagery first	Caudate	0.34	0.09	0.41	0.11	−2.07*	16
	Putamen	0.36	0.11	0.41	0.09	−1.98*	16
	Pallidum	0.36	0.12	0.40	0.12	−1.11	16
	Average	0.36	0.09	0.41	0.08	−1.80	16

Handgrip 1st *n* = 19; Mental Imagery 1st *n* = 17, * *p* < 0.05, ** *p* < 0.01. HC = healthy control, MS = multiple sclerosis, SD = standard deviation, df = degrees of freedom.

During the intrinsic alertness task for HC, the results showed significantly increased global FC, between the caudate and the putamen to the rest of the cortex, between block 1 and block 4 in the group without cognitive fatigue but showed no significant differences in the group with cognitive fatigue. Full results are displayed in Table 4.

**Table 4:** Global basal ganglia functional connectivity for the split MS groups during the intrinsic alertness task.

Task	Connectivity	Block 1		Block 4			
		Mean	SD	Mean	SD	*t* value	df
Handgrip only	Caudate	0.40	0.09	0.44	0.11	−1.05	18
	Putamen	0.42	0.10	0.41	0.10	0.21	18
	Pallidum	0.41	0.10	0.40	0.11	0.26	18
	Average	0.41	0.06	0.42	0.08	−0.74	18
Mental imagery first	Caudate	0.39	0.09	0.43	0.11	1.18*	17
	Putamen	0.39	0.08	0.41	0.09	−0.84	17
	Pallidum	0.37	0.08	0.41	0.09	1.98*	17
	Average	0.40	0.07	0.41	0.07	−0.45	17

Handgrip 1st *n* = 19; Mental Imagery 1st *n* = 18, * *p* < 0.05, ** *p* < 0.01. HC = healthy control, MS = multiple sclerosis, SD = standard deviation, df = degrees of freedom.

During the intrinsic alertness task for the MS groups, the results showed significant increased global FC between the caudate and the pallidum to the rest of the cortex, between block 1 and block 4 in the group not demonstrating cognitive fatigue but showed no significant differences in the group with cognitive fatigue, as in the HC. During the extrinsic alertness task for the MS groups, the results showed significant increased global FC between the putamen and the pallidum and the rest of the cortex between block 1 and block 4 in the group without cognitive fatigue but showed no significant differences in the group experiencing cognitive fatigue (Tables 4, 5).

**Table 5:** Global basal ganglia functional connectivity for the split MS groups during the extrinsic alertness task.

Task	Connectivity	Block 1		Block 4			
		Mean	SD	Mean	SD	*t* value	df
Handgrip only	Caudate	0.42	0.10	0.46	0.16	−1.09	18
	Putamen	0.39	0.09	0.44	0.14	−2.16*	18
	Pallidum	0.39	0.08	0.45	0.15	−2.02*	18
	Average	0.40	0.06	0.44	0.12	−1.76	18
Mental imagery first	Caudate	0.43	0.12	0.45	0.12	−0.86	17
	Putamen	0.39	0.08	0.38	0.08	0.46	17
	Pallidum	0.41	0.09	0.39	0.07	1.04	17
	Average	0.41	0.06	0.41	0.06	0.03	17

Handgrip 1st *n* = 19; Mental Imagery 1st *n* = 18, * *p* < 0.05, ** *p* < 0.01. HC = healthy control, MS = multiple sclerosis, SD = standard deviation, df = degrees of freedom.

There were no significant correlations between measures of global FC between the basal ganglia and the cortex with any measures of fatigue, subjective, cognitive or physical.

## 4. Discussion

We examined both the local and global FC of the basal ganglia to further elucidate its role in fatigue. For the local functional connectivity our results showed a significantly decreased FC connectivity between the putamen and the pallidum in the MS group compared to controls. Anatomically, the putamen forms a major input nucleus of the basal ganglia, whereas the pallidum is the major output nucleus of the basal ganglia. The basal ganglia is also strongly connected to the cortex through a number of cortical–subcortical loops (Alexander et al., 1986; Obeso et al., 2008). The dysfunctional connectivity between the putamen and the pallidum suggests a bottom-up disruption from the basal ganglia to the rest of the cortex in fatigued MS patients. Moreover, the FC between the putamen and the pallidum is negatively correlated with both subjective fatigue (FSS) and cognitive fatigue (RT) in both HC and MS groups. This indicates that the dysfunctional connectivity between the putamen and the pallidum in the MS group could underlie the fatigue associated with MS. To the best of our knowledge, no previous studies have examined the local FC of the basal ganglia during a fatigue inducing task. We are the first to provide evidence that fatigue in MS may be driven by a disruption to the FC within the basal ganglia.

To examine the effect of fatigue over the duration of the task we examined block 1 and block 4 of the task. The results demonstrated that the alertness motor paradigm successfully induced cognitive fatigue and did not produce any physical fatigue. All the groups that showed no evidence of cognitive fatigue had significantly increased global FC between the basal ganglia and the rest of the cortex. This suggests that decreased FC between the basal ganglia and the cortex may be associated with increased cognitive fatigue. This explanation would support the results of Finke et al. (2015) that showed decreased resting state FC between the basal ganglia and the medial pFC was associated with increased subject fatigue.

Interestingly, the change in FC across the task does not correlate with either subjective or cognitive fatigue, indicating that this increased connectivity may in fact represent a form of compensation. Whereby increased FC of the basal ganglia to the cortex overcomes the cognitive fatigue, to some extent. Our results do not support the conclusion of DeLuca et al. (2008). The authors mentioned that although they operationalized cognitive fatigue as increased cerebral activity, it may be a form of compensation. Our results support this alternative explanation. DeLuca et al. (2008) showed increased activity in the basal ganglia in the MS group compared to controls and concluded that this may indicate increased fatigue. However, even the MS group in the study perform better, evidenced by reduced reaction time, over the duration of the task, suggesting that this increased activity may be compensatory to maintain a practice effect. fMRI studies have shown that MS patients often exhibit increased cerebral activation compared to controls (Wishart et al., 2004; Mainero et al., 2006; Bonnet et al., 2010). Mainero et al. (2006) suggested that the altered brain activation may represent a form of functional reorganization to compensate for MS related brain changes. It is possible that this compensation indirectly effects the perception of fatigue as the task requires increased neural resources (Tartaglia et al., 2008). Given the pattern of results in the current study it is possible to posit that the local FC dysfunction between the putamen and the pallidum plays a key role in cognitive fatigue in MS, and that increased FC between the basal ganglia and the cortex is able to compensate for this fatigue to some extent.

Increased reaction time during both the intrinsic and extrinsic alertness task was significantly associated with increased subjective fatigue, which suggests that reaction time is indeed a robust measure of cognitive fatigue. Previous studies have not shown a reliable correlation between cognitive fatigue measured by performance and subjective self-report measures of fatigue. The significant correlation in the present study could indicate that tasks of sustained attention, specifically intrinsic alertness requiring internal motivation, are well suited to measuring cognitive fatigue and could help to overcome the difficulties in measuring fatigue.

The results of the current study suggest that the local FC between the putamen and the pallidum could serve as a neurophysiological biomarker of fatigue. The FC connectivity between these regions during a fatigue inducing task could be used in clinical practice to assess the effectiveness of interventions for fatigue and help establish a more systematic way of measuring fatigue.

It is important to note that in the present study we only included females. This was due to two reasons, (1) there are a large amount of sex differences in the attention literature (Der and Deary, 2006; Jain et al., 2015; Riley et al., 2016), and (2) MS affects significantly more females then males, with an approximate ratio of 3:1 (Wallin et al., 2019). However, future studies may be able to include a balanced sample of males and females to determine whether there is a sex plays a role in fatigue or the functional connectivity of the basal ganglia. In addition, future studies could examine whether a similar functional connectivity disruption in the basal ganglia exists at rest, as this may suggest an inherent dysfunction of the basal ganglia connectivity in MS fatigue. Future studies could also investigate whether there is an association between functional connectivity and performance measures of fatigue and inflammatory or immune disease markers (e.g., IL-6). This study provides a better understanding of the neural mechanisms that may underlie fatigue in MS. Further work is required to develop treatments that may target these mechanisms. Functional connectivity changes could potentially be biomarker to examine the effectiveness of interventions/treatments for fatigue in MS.

### 4.1. Conclusion

Our results further evidence that the basal ganglia is a key neural substrate of cognitive fatigue. We extend the current knowledge of the basal ganglia’s involvement in cognitive fatigue to suggest that the decreased local FC within the basal ganglia may be a driving factor for fatigue. Increased global FC between the basal ganglia and the cortex may subserve a compensatory mechanism to reduce the impact of cognitive fatigue. Given the complexity of measuring fatigue a more systematic measurement is required. The current study provides evidence that tasks of internal motivation are most suited to measuring fatigue. Moreover, the local FC of the basal ganglia during fatigue inducing tasks could provide a neurophysiological biomarker of fatigue.

## Data availability statement

The datasets presented in this article are not readily available because the participants and ethical committee of the study did not give consent for the data to be shared. Requests to access the datasets should be directed to cl798@medschl.cam.ac.uk.

## Ethics statement

The studies involving human participants were reviewed and approved by Frenchay Research Ethics Service (16/SW/0059). The patients/participants provided their written informed consent to participate in this study.

## Author contributions

CL was involved in data collection, data analysis, and writing of the manuscript. NM was involved in study supervision, data analysis, and editing of the manuscript. SG was involved in data analysis and editing of the manuscript. GM was involved in study conception, study supervision, and editing of the manuscript. AS was involved in participant recruitment and editing of the manuscript. RJ was involved in study conception, participant recruitment, and editing of the manuscript. JB was involved in study supervision and editing of the manuscript. NT was involved in study conception, study supervision, data analysis, and writing of the manuscript. All authors contributed to the article and approved the submitted version.

## Funding

This study was supported by Multiple Sclerosis Research, Treatment and Education (Registered Charity Number 1043280, Company No. 3005230).

## Conflict of interest

The authors declare that the research was conducted in the absence of any commercial or financial relationships that could be construed as a potential conflict of interest.

## Publisher’s note

All claims expressed in this article are solely those of the authors and do not necessarily represent those of their affiliated organizations, or those of the publisher, the editors and the reviewers. Any product that may be evaluated in this article, or claim that may be made by its manufacturer, is not guaranteed or endorsed by the publisher.

## Footnotes

^1^ http://www.fil.ion.ucl.ac.uk/spm
